# Wildfire Smoke and Human Health: A Global Perspective on Respiratory Risks and Emerging Interventions

**DOI:** 10.1021/envhealth.6c00112

**Published:** 2026-04-23

**Authors:** Patricia Krecl, Caradee Y. Wright, Sara Basart, Carlyn J. Matz, Daniel Tong, Jennifer D. Stowell, Mikhail Sofiev

**Affiliations:** † Graduate Program in Environmental Engineering, 74354 Federal University of Technology, Av. dos Pioneiros 3131, 86036-370 Londrina, Brazil; ‡ Climate Change and Health Research Programme, Environment and Health Research Unit, South African Medical Research Council, Pretoria 0084, South Africa; § Department of Geography, Geoinformatics and Meteorology, University of Pretoria, Pretoria 0002, South Africa; ∥ World Meteorological Organization (WMO), 1201 Geneva, Switzerland; ⊥ Water & Air Quality Bureau, Health Canada, Ottawa, ON K1A 0K9, Canada; # Atmospheric, Oceanic and Earth Sciences Department, George Mason University, Fairfax, Virginia 22030, United States; ∇ Department of Global, Environmental, and Occupational Health, University of Maryland School of Public Health, College Park, Maryland 20742, United States; ¶ Finnish Meteorological Institute, Atmospheric Composition Department, Helsinki FI-00560, Finland

The frequency and intensity of wildfires in many regions over the globe,^1^ largely driven by anthropogenic impacts, land-use changes, and urban expansion, and facilitated by climate change, have brought a new dimension to global health. Beyond the immediate destruction of ecosystems and property, wildfire smoke has emerged as a pervasive hazard with far-reaching impacts on respiratory health. From North and South America to Australia and southern Europe, studies converge on the strong associations between wildfire smoke exposure and adverse health outcomes. Yet the evidence on effective health interventions remains nascent. This Viewpoint summarizes global thinking on wildfire exposure and human health, highlighting the gaps in implemented interventions, and reflecting on societal and scientific directions urgently needed to protect vulnerable populations.

There is growing evidence of respiratory wildfire-related health impacts across diverse regions (e.g., Australia, Brazil, Canada, Spain, and USA) underscoring the global relevance of this hazard.^2^ Systematic reviews (e.g., ref 3) demonstrate a strong and consistent association between wildfire smoke exposure, particularly fire-induced fine particulate matter, and respiratory morbidity. Reported outcomes include increases in asthma and chronic obstructive pulmonary disease (COPD) exacerbations, resulting in increased emergency department visits, hospital admissions, and respiratory medication dispensation (e.g., ref 4). Furthermore, experimental studies using cell lines and animal models provide mechanistic insight into the observed health effects.^5^

There are multiple vulnerable populations at highest risk from wildfire smoke exposures. Children under five years, the elderly, pregnant women, the unborn fetus and people with pre-existing conditions (i.e., asthma, COPD, cardiovascular disease) consistently emerge as susceptible groups.^6^ Social vulnerability also compounds risk, including those related to lower socioeconomic status or racial/ethnic minorities, which often exhibit disproportionately higher impacts.^7^

While studies of respiratory morbidity dominate the evidence, there is increasing recognition of broader health effects that go beyond the lungs. Premature death, cardiovascular morbidity, neurological impacts and fetal outcomes have all been reported, indicating that wildfire smoke is not merely a transient respiratory irritant but a multisystemic stressor.^3^

About 90% of wildfires are caused by humans, either deliberately or accidentally where agriculture and industry practices, land use conversion (e.g., clear-cut of forests) as well as recreational activities are among the most common causes.^8^ A special type of fires are prescribed burns, which are used in many regions to prevent devastating fire events by reducing the fuel and cleaning out the flammable debris. Proper planning of these fires accounting for meteorological conditions can significantly reduce population exposure to wildfire smoke compared to a large, catastrophic fire; however, prescribed fires also produce smoke and the trades-offs should be considered.^9^ Prescribed fires could present prime opportunities for clinical interventions since these types of fires tend to be planned in advance and can provide clinicians with opportunity to educate patients and provide prophylactic care when necessary. Natural wildfires can be triggered by lightning and peat burning, but many are often exacerbated by forestry practices and management.^10^ Additionally, wildland-urban interface (WUI) fires —which are wildland fires that spread into communities— increased by 23% in number and 35% in burned area in the period 2005–2020, driven by climate change.^11^ WUI fires are of particular concern given the burning of synthetic materials not normally found in natural wildfire smoke.^12^ Reducing population exposure during WUI fires can be primarily achieved through proactive planning, robust evacuation systems, and community preparedness.

In terms of public health and behavioral strategies during an ongoing or approaching fire smoke event, risk communication remains the most common strategy: advisories to stay indoors, limit outdoor activity, and/or adjust medication use during the episodes.^13^ However, few of these strategies have been evaluated for effectiveness. For example, parents in wildfire-affected regions self-initiated measures such as keeping children indoors and increasing asthma medication, but no formal evaluation was undertaken.^6^ More research is needed regarding the effectiveness of these measures. Often, wildfires occur during periods of high temperatures, and not all individuals have access to protective measures like air conditioning.^14^ Thus, staying indoors on a wildfire smoke day may contribute to heat-related health effects or little differences between indoor and outdoor temperatures. More recent proposals emphasize scaling up such cleaner-air shelters^15^ and distributed indoor filtration systems,^16^ but implementation lags behind urgency.^17^ In many countries, advice to “stay indoors” might be impractical in overcrowded, poorly ventilated housing.

At the science-society interface, health inequities are amplified. Wildfire smoke does not impact all equally. The majority of the evidence is from high-income countries such as Australia, Canada, and USA, while less is known about the risks in populations in low- and middle-income countries, where health systems are fragile and air quality monitoring systems sparse.

Advances in atmospheric composition monitoring (including satellite-based smoke tracking and in situ networks) and modeling have improved detection and forecasting of wildfire smoke. Smoke pollution forecast systems integrate satellite data and meteorological models to predict the movement and concentration of hazardous smoke pollution plumes (including particulate matter and reactive gases).^18^ However, forecasts face challenges due to the rapid evolution of fires and the complex chemistry of smoke. Furthermore, climate research focuses on incorporating climate indicators into subseasonal-to-seasonal (i.e., weeks to months ahead), annual and decadal predictions^19,20^ to improve operational fire risk management. These systems aim to incorporate information from remote ocean teleconnections (like El Niño/La Niña) and local climate data streams to provide early warnings of heightened fire potential. A key challenge is seamlessly integrating various data types and overcoming model limitations at local scales. However, the translation of this atmospheric composition exposure information into community-level health interventions remains slow. Some exceptions are countries (e.g., Canada and USA) that already have health-based air quality alerting systems, meaning that the impacted population know that the outdoor air quality is poor and they should alter behaviors. Future directions include randomized trials of filtration interventions, integration of wildfire modules into electronic health systems for real-time tracking, and inclusion of psychosocial outcomes in studies of the health effects.

Looking forward, the global evidence is clear: wildfire smoke is a significant and growing health hazard in many regions. Advanced smoke pollution warning systems are required, but they are oftentimes hampered by the lack of comprehensive in situ monitoring infrastructure, especially in low- and middle-income countries (e.g., refs 21, 22 ). National Meteorological and Hydrological Services are critically involved in all stages of the smoke pollution response process, from early warning to real-time incident support, by providing the essential weather data and models that drive smoke dispersion forecasts while responsibility for responding to the health impacts of smoke pollution falls on a network of public health organizations, environmental protection agencies, and fire management services at local, national, and international levels. The next frontier in population health is implementation science—rigorously testing interventions that are context-appropriate, scalable, and equity-sensitive. By integrating health perspectives into wildfire management and climate adaptation strategies, societies can better prepare for a future where smoke seasons may become as predictable—and as dangerous—as heatwaves or floods. Protecting air quality is no longer a luxury of clean environments but a necessity for resilient health systems worldwide.
